# Sexual Dysfunction in Type 2 Diabetes at Diagnosis: Progression over Time and Drug and Non-Drug Correlated Factors

**DOI:** 10.1371/journal.pone.0157915

**Published:** 2016-10-05

**Authors:** Giovanni Corona, Carlo B. Giorda, Domenico Cucinotta, Piero Guida, Elisa Nada, SUBITO-DE Study Group

**Affiliations:** 1 Endocrinology Unit, Major Hospital, Bellaria, Bologna Italy; 2 Metabolism and Diabetes Unit, ASL Turin 5, Chieri Italy; 3 Department of Medicine Policlico of Messina, Messina Italy; 4 Statistical Consultant for Associazione Medici Diabetologi, Rome, Italy; 5 Chaira Medica Association, Chieri, Italy; Old Dominion University, UNITED STATES

## Abstract

**Aims:**

To present the longitudinal data of the SUBITO-DE study, a prospective survey involving male patients with new or recently diagnosed type 2 diabetes mellitus (T2DM) (<24 months).

**Materials and Methods:**

Sexual function was assessed in male patients with T2DM at baseline (phase 1) and after a mean follow-up of 18 months (phase 2). Standard metabolic parameters and sexual and depressive symptoms were evaluated.

**Results:**

Six of the 499 enrolled patients died of different causes during phase 1. Of the 493 surviving men invited to participate in phase 2, 450 (mean age 59.0±9.0 years) (90.2%) accepted and 43 (8.2%) were lost to follow-up. As compared to baseline, the proportion of the men who reported improvement in erectile dysfunction (ED) at follow-up was nearly double that of the men who reported worsening of ED (22.6% vs. 12.8%). The increase in frequency of sexual activity the men reported at follow-up assessment indicates that many never treated before baseline were taking an ED drug during the study period (106 subjects). Phosphodiesterase type 5 inhibitors (PDE5i) were the ED drugs most commonly taken at both baseline and follow-up. An overall improvement over baseline values was observed in metabolic targets for T2DM and depressive symptoms. Conversely, no change in lifestyle behaviors was recorded during the study.

**Conclusions:**

Sexual dysfunction is a major concern in men with T2DM. The SUBITO-DE study demonstrates that, when combined with adequate counseling and tailored PDE5i therapy, an integrated approach to achieving metabolic targets in men with T2DM can improve sexual function as well as depressive symptoms.

## Introduction

Epidemiological studies worldwide have documented that erectile dysfunction (ED) is one of the major complications of diabetes mellitus (DM) in men. [1–6] Early diagnosis of ED and the identification of its risk factors, particularly in men with type 2 DM (T2DM), can provide useful information for stratifying cardiovascular risk as well. A recent meta-analysis of 12 studies demonstrated that ED is associated with a substantial increase in the risk of cardiovascular events, coronary heart disease, and peripheral vascular diseases. [7] Risk was found to be independent of age, metabolic control, body-mass index (BMI, weight in kg divided by height in meters squared), and duration of the condition, emphasizing the importance of ED screening and its early detection in the diabetic male population. [7]

Despite this evidence, the presence of ED in diabetic men is still poorly evaluated in routine clinical practice for several reasons. Men with ED, and particularly those with T2DM, are reluctant to report the condition to their doctor. [8] Moreover, physicians have identified multiple barriers to managing ED in their practice, [8–9] making it difficult even for healthcare professionals to enquire about ED in routine diabetes care. [10] In line with these data, a previous Danish study showed that only 33% of men with T2DM reported that their general practitioner had brought up sexuality in the consultation. [11] Similar findings from a study by Bjerggaard et al. [12] showed that about half of sexually inactive men with T2DM felt that their sexual life did not meet their sexual needs; the study also found that sexual distress was more common among sexually inactive than sexually active men. [12]

The true incidence of ED in the male diabetic population remains largely unknown. According to a large-scale study conducted by Fedele et al. [8] and involving 1010 male diabetic patients, the crude incidence of ED at a mean follow-up of 2.8 years was 68 cases per 1000 person-years, more than two-fold that estimated in the general population. A higher incidence (166.3 per 1000 person-years) was reported by De Berardis et al. [9] in another Italian survey that enrolled 670 men with T2DM followed every 6 months for up to 3 years. Conversely, a lower incidence (25/1000 person-years) was reported by Klein et al. [10] in a 10-year cumulative U.S. study in 264 type 1 DM men who were less than 30 years of age at diagnosis of diabetes.

T2DM is a costly disease affecting approximately 6.5% of native adults in Italy. [13] Treatment can prevent some of its devastating complications but does not usually restore normoglycemia or eliminate the adverse consequences altogether. [14–15] Since the current methods of treating diabetes remain inadequate, prevention is essential for early diagnosis in at-risk populations and to reduce its chronic complications. Lifestyle changes and treatment with metformin have been found to reduce the incidence of diabetes in persons at high risk. [15] Similarly, control of comorbidities and lifestyle modifications are associated with improvements in ED. [16]

Epidemiological data can inform prevention strategies and allocation of adequate resources. We previously reported the prevalence of ED and its correlates in a sample of male patients with new or recently diagnosed T2DM (<24 months) attending diabetes care centers affiliated with the Association of Medical Diabetologists (AMD; the SUBITO-DE study). [17–18] We extended our analysis and now present longitudinal data from the same study after a mean follow-up of 18 months.

## Materials and Methods

### Study design

The SUBITO-DE study is an observational, multicenter, cross-sectional prospective study involving diabetes care centers located throughout Italy and selected according to the efficiency indicators outlined in the AMD Foundation Quality Manual. The study was performed between May 2010 and December 2011 (phase 1) and included 499 patients (final enrolment rate 33.3%). [17–18] Follow-up (phase 2) assessment started in November 2011 and concluded in August 2013, comprising a period of about 18 months. The study protocol and the consent procedure were approved by the local ethics committee at each participating center and the subjects were evaluated using the same instruments and methods as in phase 1 data collection. [17–18] All participants provided their written informed consent to participate in this study.

### Sample selection

Without preliminary selection, all male patients recently (<24 months) diagnosed with T2DM were consecutively interviewed by their attending physician at their diabetes care center and asked whether they had experienced a change in sexual function or found it unsatisfactory. Those responding positively were then invited to participate in the study (baseline, phase 1). The only exclusion criteria were inability to understand the purpose of the study or to respond to the questionnaire items, and limited life expectancy due to severe systemic disease. The responses of all interviewees were recorded; only those patients who had given their informed written consent to participate were included in the study. A diagnosis of T2DM was established according to the 2009–2010 guidelines of the Italian Standards of Care for Diabetes Mellitus. [19]

### Follow-up visit

After a mean follow-up of 18 months, the subjects enrolled in phase 1 were invited to participate in phase 2 of the study. Follow-up assessment was identical to that performed at baseline (phase 1). Briefly, the interviews were conducted according to standard methods for collecting the general characteristics of the patient and diabetic disease.

ED was evaluated and graded according to the erectile function domain of the International Index of Erectile Function (IIEF), a multidimensional scale for the assessment of ED. [17–18] The unit for defining improvement and worsening of ED at phase 2 was a subject who had moved up or down in IIEF class between phase 1 and phase 2. Depressive symptoms were graded by means of the Center for Epidemiologic Studies Depression Scale (CES-D), a self-report depression scale for research in the general population. [17–18] ANDROTEST, a structured interview for screening hypogonadism in patients with sexual dysfunction, was used to assess symptoms suggestive of hypogonadism. [20] ANDROTEST is a 12-item interview previously validated for the screening of hypogonadism in patients with sexual dysfunction. [20–22] Responses are assigned a score of 0 to 3 on a Likert scale by the interviewer. The total score ranges from 1 to 32. A high score identifies a higher prevalence of hypogonadism-related signs and symptoms. [20–21] For example, a score > 8 is prognostic for low testosterone, with a sensitivity and specificity of about 70%. [20–21]

All patients underwent complete clinical examination. No clinical and/or laboratory tests were performed other than those recommended by the guidelines of the Italian Standards for Care of Diabetes Mellitus [19] and international societies of urology and andrology. [23–25] Hypogonadism was defined according to the criteria of international guidelines [23–25] and those proposed by the European Male Aging Study (EMAS): the presence of at least three sexual symptoms (erectile dysfunction, decreased libido, and nocturnal erections) associated with total testosterone <2.8 ng/mL. [26]

### Statistical analysis

The data are presented as the mean ±standard deviation; categorical variables are described as frequencies and percentages. The aim of analysis was to describe the characteristics of the patients participating in both study phases. Intra-patient comparisons of measurements taken at the two assessments were performed using paired Student’s t-test and the non-parametric Wilcoxon signed-rank test when appropriate. Comparisons between proportions were performed using McNemar's test. Baseline characteristics of patients lost to follow-up and those who completed the study were compared using Student's t-test for independent samples and chi-squared or Fisher's exact test for the categorical variables. Statistical analyses were performed using STATA software, Version 12 (StataCorp, College Station, TX, USA). P values <0.05 were considered statistically significant.

## Results

Six (1.2%) of 499 patients died of different causes between phase 1 and 2. Of the 493 surviving men invited to participate in the second phase, 450 (90.2%) (mean age 59.0±9.0 years) accepted, 43 (8.2%) of which were subsequently lost to follow-up. Table 1 presents the clinical and biochemical characteristics of the patients assessed at follow-up versus baseline.

**Table 1:** Descriptive characteristics of the study population.

Variable	Baseline	Follow-up	p-value
***Lifestyle (%)***			
Former smoker	36.4	37.0	0.439
Smoker	30.0	30.4	0.617
Coffee	89.6	76.9	<0.001
Alcoholic beverages	64.4	64.4	1.000
Occasional physical activity	38.4	40.0	0.463
Regular physical activity	30.4	27.8	0.128
***Associated conditions and chronic complications (%)***			
Arterial hypertension	55.8	59.1	-
Dyslipidemia	40.9	46.4	-
Coronary heart disease	4.9	6.4	-
Myocardial infarction	2.7	2.9	-
Diabetic retinopathy	7.1	8.9	-
Diabetic nephropathy	12.9	18.4	-
Diabetic neuropathy	8.7	11.1	-
***Use of diabetes medications (%)***			
Sensitizer*	68.2	73.6	0.002
Insulin secretagogue	20.4	20.7	0.882
Insulin	10.9	10.7	0.853
Incretin**	13.3	23.6	<0.001
ACE-inhibitors	27.1	28.0	0.527
ATII antagonists	24.4	28.7	0.006
Beta-blockers	17.1	19.6	0.034
Calcium antagonists	13.8	13.8	1.000
Diuretics	20.2	24.2	0.008
Nitrates	2.0	0.7	0.014
Statins	37.1	42.4	0.011
Ezetimibe	3.8	5.6	0.033
Fibrates	2.2	3.8	0.020
Antiplatelet agents	23.6	29.1	<0.001
Antithrombotics	6.9	4.0	0.009
***Use of ED drugs (%)***	21.6	38.0	<0.001
Regular:			
	2.0	2.2	
*-Sildenafil*			
*-Vardenafil*	1.1	5.3	
*-Tadalfil*	1.8	2.7	
*-PGE1*	0.0	0.2	
*-Testosterone*	0.0	0.0	
Occasional:		6.7	
*-Sildenafil*	9.1		
*-Vardenafil*	3.8	10.4	
*-Tadalfil*	5.6	7.8	
*-PGE1*	1.1	0.7	
*-Testosterone*	0.2	0.2	
***Reason for abandoning PDE5i therapy***	62.5%***	33.3%***	
High cost:		15.0 (n = 40)	
*-Sildenafil*	28.0 (n = 50)		
*-Vardenafil*	27.3 (n = 22)	15.5 (n = 71)	
*-Tadalfil*	12.1 (n = 33)	23.4 (n = 47)	
Therapeutically ineffective:		27.5 (n = 40)	
*-Sildenafil*	38.0 (n = 50)		
*-Vardenafil*	31.8 (n = 22)	12.7 (n = 71)	
*-Tadalfil*	30.3 (n = 33)	17.0 (n = 47)	
***Clinical data***			
*Anthropometric parameters*			
Body-mass index (kg/m^2^), n = 449	29.3±4.7	29.1±4.5	0.045
Abdominal circumference (cm), n = 433	104±13	103±12	0.231
Systolic blood pressure (mm Hg), n = 443	132±15	131±12	0.016
Diastolic blood pressure (mm Hg), n = 443	80±9	79±7	0.284
Heart rate (beats per minute), n = 414	76±7	75±6	0.175
*Biochemical parameters*			
Glycated hemoglobin (%), n = 443	7.2±1.5	6.9±1.1	<0.001
Total cholesterol (mg/dl), n = 419	186±40	176±36	<0.001
HDL cholesterol (mg/dl), n = 398	46±13	46±12	0.876
LDL cholesterol (mg/dl), n = 378	111±35	103±32	<0.001
Triglycerides (mg/dl), n = 416	154±82	147±85	<0.001
Creatinine (mg/dl), n = 383	0.96±0.20	0.99±0.40	0.134
Uricemia (mg/dl), n = 286	6.2±5.2	5.6±1.4	0.049
ALT (U/L), n = 370	27±15	27±20	0.905
AST (U/L), n = 370	28±16	28±22	0.717
Total testosterone (ng/ml), n = 254	4.3±3.2	4.1±2.2	0.182
Total testosterone (ng/ml) <2.31, n = 254	24.0	12.6	<0.001
Total testosterone (ng/ml) <3.5, n = 254	47.6	42.9	0.146
EMAS criteria, n = 198	16.2	12.1	0.144
*Depressive symptoms*			
CES-D score	16±9	14±9	0.001
Suspected depression	18.7	14.9	0.035

Plus-minus values are the means ±SD; categorical data are given as percentage. ED denotes erectile dysfunction, BMI body-mass index, EMAS European Male Aging study, CES-D Center for Epidemiologic Depression Scale.

* metformin or pioglitazone and no segretagogues

** any DPP IV inhibitors or GLP-1 agonists

*** At least one PDE5ì abandoned.

Table 2 shows the distribution of patients according to ED severity at baseline and at follow up. A total of 101 (22.4%) of 450 patients reported sexual inactivity in at least one of the two visits (46 at both visits, 36 only at baseline, and 19 at follow-up). At baseline 82 patients (18.2%) stated they were sexually inactive; 65 (14.4%) reported not having had sexual intercourse in the 4 weeks prior to follow-up assessment. Among the 450 patients, 58 (12.9%) reported worsening of ED as compared with their condition at baseline. The latter prevalence increased to 16.6% when the sexually inactive patients were excluded from the analysis. Conversely, 102 (overall 22.7% and 29.2% when only sexually active men were considered) subjects reported an improvement in ED.

**Table 2:** Prevalence of erectile dysfunction (ED) severity based on the International Index of Erectile Function (IIEF) short version score (No ED >21; Mild ED 17–21, Mild-moderate ED 12–16, Moderate ED 8–11, severe ED <8) at baseline (phase 1) and follow-up assessment (phase 2) in the whole study population and after excluding subjects who reported no sexual activity.

**Whole study population**	**Phase 1 n (%)**	**Phase 2 n (%)**
No sexual activity	82 (18.2)	65 (14.4)
Severe ED	20 (4.4)	14 (3.1)
Moderate ED	41 (9.1)	33 (7.3)
Mild-Moderate ED	68 (15.1)	55 (12.2)
Mild ED	89 (19.8)	87 (19.3)
No ED	150 (33.3)	196 (43.6)
Total	450	450
**Excluding subjects who reported no sexual activity**	**Phase 1 n (%)**	**Phase 2 n (%)**
Severe ED	18 (5.2)	11 (3.2)
Moderate ED	39 (11.2)	29 (8.3)
Mild-Moderate ED	64 (18.3)	51 (14.6)
Mild ED	84 (24.1)	77 (22.1)
No ED	144 (41.3)	181 (51.9)
Overall ED improvement	-	102 (29.2)*
Overall ED worsening	-	58 (14.9)
Total	349	349

*p<0.001 vs. worsening ED at follow-up. The unit defining improvement and worsening of ED was a subject who had moved up or down in IIEF class between phase 1 and phase 2.

### Lifestyle

No changes in smoking, alcohol consumption or frequency of physical activity were observed during the entire study period. Conversely, as compared with the rate reported at baseline, a lower proportion of patients said they drank at least 1 cup of coffee daily (Table 1).

### Associated illnesses and chronic complications

At follow-up assessment, 7 (1.6%) patients reported having experienced a coronary ischemic event; arterial hypertension and dyslipidemia were newly diagnosed in 15 (3.3%) and 25 (5.6%) patients, respectively. As expected, a higher proportion of chronic diabetic complications was also recorded at follow-up.

### Use of diabetes medications

In comparison with baseline, a higher proportion of patients at follow-up assessment were receiving treatment with insulin sensitizers and incretins, the latter being the most commonly used oral hypoglycemic drug. In addition, the use of angiotension II receptor blockers (ATII antagonists) and other cardiovascular-related medicines, including beta blockers, antiplatelet drugs, and lipid-lowering agents in particular, was higher. Conversely, the rate of use of nitrates and antithrombotic drugs was lower (Table 1).

### Use of ED medications

During the period between the two visits, 171 (38.0%) patients used ED drugs. Among those never treated before baseline, 106 subjects were taking an ED drug during the study period. Phosphodiesterase type 5 inhibitors (PDE5i) were the most commonly used ED drugs during the study. Sildenafil was the most frequent PDE5i reported at baseline and vardenafil the most common at follow-up assessment. As compared with the rates reported at baseline, fewer patients at follow-up reported they had abandoned taking any PDE5ì. Inefficacy was claimed the most common reason to abandon PDE5i at baseline. Conversely, the high cost of PDE5i was the most frequent motive given at follow-up for discontinuing its use (Table 1).

### Clinical data

A small but marked reduction in BMI and systolic blood pressure was observed at follow-up. There was a greater reduction in total cholesterol and triglyceride levels but no change in high-density lipoprotein (HDL) levels. Both glycated hemoglobin and uricemia levels improved markedly but no changes in the other biochemical parameters or testosterone levels were observed. However, there was a lower rate of severe biochemical hypogonadism (total testosterone < 2.31 ng/mL). Finally, there was an appreciable improvement in depressive symptoms, as measured by CES-D scores, with a lower proportion of patients with a CES-D score >24 suggesting clinical depressive symptoms (Table 1).

### Lost to follow-up

There was no difference in age, lifestyle behaviors or associated morbidities or chronic complications between the patients assessed at follow-up and those lost to follow-up (see supplementary Table 1). In the patients lost to follow-up, the use of insulin sensitizers and statins was lower; there was no difference in the use of any of the other drugs, including ED medications. The clinical characteristics were substantially similar between the two groups, except that the patients lost to follow-up were noted to have markedly higher creatinine levels and CES-D scores (see S1 Table).

## Discussion

Here we describe for the first time the management and follow-up of men with ED and T2DM at high risk of developing cardiovascular disease. Our study shows that, as compared with baseline, the proportion of men who reported an improvement in ED at follow-up assessment was nearly double that of the men who stated that their sexual performance was poorer. However, in this relatively young sample of men with recently diagnosed T2DM, about 30% reported no recent sexual activity at baseline and follow-up assessment. This finding might reflect the fact that diabetic men are generally reluctant to report the problem to their doctors [8–9] and that healthcare professionals are hesitant to screen for ED during routine practice. [10] Whatever its causes, lack of sexual activity has important clinical implications. The frequency of sexual activity figures among the lifestyle factors that promote longevity. [27–28] A recent longitudinal study of a sample of 1687 men seeking medical care for ED and followed for a mean follow-up period of 3–4 years showed that a higher frequency of sexual intercourse significantly reduced the risk of major adverse cardiovascular events (MACE), even after adjusting for known cardiovascular risk factors. [29] These findings are shared by several other epidemiological studies which suggested health benefits derived from sexual activity, as demonstrated by an inverse relationship between male sexual activity and mortality, with a favorable effect of relational intimacy and sexuality on overall and cardiovascular health. [30–32] Frequent sexual activity with the same partner may create a better supportive intimate relationship, reduce stress, and increase social support, ultimately enhancing cardiovascular health. Like any other kind of physical exercise, sexual activity may have protective functions. [29] Talib et al. [33] recently reported that increased frequency of sexual activity after penile prosthesis implantation was significantly associated with an improvement in glycated hemoglobin levels.

Involvement of the partner plays a key role in the evaluation and management of patients with ED. [34–35] There is consistent evidence that women engage less frequently in sexual activity after their partner develops ED and that their sex life is less satisfactory when ED is severe. [36–50] Furthermore, women whose partners have ED retrospectively reported a substantial decline in their own sexual desire, sexual arousal, orgasm and satisfaction after their partner had developed ED. [36–50] Of note, however, is that female sexual dysfunction such as dyspareunia or vaginal dryness can exacerbate milder forms of ED. [36–50]

Despite general satisfaction with the use of PDE5i, the discontinuation rate ranges from 14 to 80%. [51–52] The reasons for this are not completely clear. It may ensue from a complex interplay between treatment efficacy and satisfaction, side effects and safety concerns, cost, and psychological factors. [51–52] The high cost of prescription refills is often claimed as the major reason patients abandon PDE5i treatment according to some studies [53] but not to others. [54–55] In our study, more patients on tadalafil said they abandoned the drug due to its cost. Poor adherence to treatment with PDE5is may be partially explained by the inability of physicians to tailor the treatment to the patient’s needs, which highlights inaccurate drug selection. [51] Moreover, pharmacological treatment alone does not resolve the many different problems manifesting in ED, including anxiety, loss of self-esteem, depressed mood, poor communication between the couple or the partner’s sexual dysfunction. [51–55] Hence, involving the patient in deciding on treatment options, in combination with adequate counseling and follow-up, are the cornerstones for successful ED medical treatment.

The SUBITO-DE study was developed and carried out also with this aim in mind. The present data document that a dedicated approach to diabetic men with ED can significantly increase the use of PDE5i, reduce the dropout rate, and improve overall sexual function. While therapeutic ineffectiveness was the most common reason given at baseline for discontinuing treatment with PDE5i, the cost of prescription refills was the most frequent reason for discontinuation given at follow-up assessment, underscoring the importance of counseling to improve satisfaction with PDE5i efficacy. [56–57] The low number of patients lost to follow-up and, in particular, the absence of marked differences in the main clinical parameters between the patients who presented at follow-up and those lost to follow-up, indicate that sexual function is a major component in the well-being of diabetic men. A recent large-scale survey of diabetic patients in China found that more than 75% of the subjects interviewed wished to receive management for their sexual problems. [58] Similar results were previously reported by other authors. [52]

Recognizing ED and its role as a possible further risk factor for cardiovascular disease might motivate diabetic men to improve their metabolic control and treatment adherence. While the present data cannot clarify this point, it is interesting to note that, despite only a slight modification in lifestyle behaviors, an overall improvement in glycated hemoglobin was observed at follow-up assessment. Whether this finding can be attributed to an improvement in sexual function *per se* or is a consequence of a different diabetic medical approach is beyond the scope of the present study. What is important to recognize, however, is that besides sexual function, an improvement in depressive symptoms was also observed at follow-up. The association between ED and depression is well known, [59–61] and depressive symptoms were one of the most determinant factors for moderate-severe ED at baseline in the SUBITO-DE study. [18] T2DM is far more common in people with major depressive disorders, [62–63] and depressive symptoms can impair diabetic control by negatively influencing diet and adherence to medical therapy. Also, depressive symptoms are inversely related to testosterone levels. [20, 64] The reduction in the prevalence of severe hypogonadism at follow-up assessment might have contributed to the observed improvement in depressive symptoms.

Another finding that merits attention is the therapeutic targets achieved in the study sample. As compared with baseline, an overall improvement in all major metabolic targets was observed at follow-up: even the average low-density lipoprotein (LDL) and triglyceride levels, outside the target levels at baseline, were within the range recommended by international societies and the Italian Standards of Care for Diabetes Mellitus. This is reflected by the increase in the use of lipid-lowering drugs in the patients assessed at follow-up. The improvement in lipid profiles, and the increased use of statins in particular, could have independently contributed to the improvement in sexual function reported at the follow-up assessment. A recent meta-analysis of the available evidence documented that statins can lead to a clinically relevant improvement in erectile function, as measured by the five-item version of the IIEF. [65] Finally, the present data indicate a greater improvement in testosterone levels in the severe hypogonadal men than in those with mild hypogonadism. There is evidence for an inverse relationship between BMI and testosterone levels [66–69] but the reasons why the two are related are complex and not completely understood. A previous meta-analysis documented that body weight loss, whatever the amount achieved, was associated with an improvement in testosterone levels. [70] In our study, the overall improvement in BMI noted at follow-up assessment might at least partially explain the reduction in severe hypogonadism and the improvement in uric acid levels as well. A recent meta-analysis documented that testosterone replacement therapy (TRT) may improve body composition and glycemic control in hypogonadal men from the general population. [68] Similar data have been reported in T2DM, [70–72] and TRT has been shown to improve all aspects of sexual function. [73] Though no patients in our study were receiving TRT, screening for and treatment of hypogonadism in patients with T2DM certainly seems advisable.

This study has several limitations. The study population was patients attending Italian diabetes care centers who may differ from those seen by general practitioners or not seeking medical care at all. Furthermore, the results obtained from specific clinical settings cannot be easily generalized to wider populations. Conversely, the phenomena observed in samples from the general population cannot always be extended to patients seeking treatment for a specific condition.

In conclusion, our findings share previous observations that sexual function is a major concern for men with T2DM. The SUBITO-DE study provides evidence that, when combined with adequate counseling and a tailored PDE5i therapy, an integrated approach to helping men with recently diagnosed T2DM achieve their metabolic targets may also improve sexual function and depressive symptoms.

## Supporting Information

S1 Appendix (DOC)

S1 Table Baseline characteristics of patients lost to follow-up. (DOC)
