# Supplementary material for: Sexual Dysfunction in Type 2 Diabetes at Diagnosis: Progression over Time and Drug and Non-Drug Correlated Factors
Source: PLoS One. 2016 Oct 5;11(10):e0157915. doi: 10.1371/journal.pone.0157915 (PMC5051725; doi:10.1371/journal.pone.0157915)
Supplement: S1 Appendix — (DOC) [file pone.0157915.s001.doc]

*SUBITO-DE Study Group

Aglialoro A., Albanese V., Albano S., Antonangelo C., Baccetti F., Bulzomi’ R., Calatola P., Capano F., Clemente G., Corigliano G., Corona G., Cucinotta D., De Fazio C., De Francesco C., De Joannon U., Del Buono A., Fontana L., Fornengo R., Fraticelli E., Gaviglio D., Gentile S., Giorda C. B., Giorgianni L., Guarino G., Iannarelli R., Improta L., Improta M., Leotta S., Magro G., Maiani L., Mastroiacovo D., Mingardi R., Morviducci L., Nada E., Nosso G., Nuzzi A., Ocelli C., Paciotti V., Pata P., Rampini P.A., Rovere M., Sabbatini A., Sciarrafia M., Sciarretta F., Sforza A., Starnone V., Testori G., Trevisan F., Turco S., Viviani G., Zavaroni D.
