# Supplementary material for: Sexual Dysfunction in Type 2 Diabetes at Diagnosis: Progression over Time and Drug and Non-Drug Correlated Factors
Source: PLoS One. 2016 Oct 5;11(10):e0157915. doi: 10.1371/journal.pone.0157915 (PMC5051725; doi:10.1371/journal.pone.0157915)
Supplement: S1 Table — (DOC) [file pone.0157915.s002.doc]

**Supplementary Table 1.** Baseline characteristics of patients lost to follow-up. *The p-values refer to the comparison of baseline characteristics between patients lost to follow-up and those who completed both phases of study as reported in Table 1.

|  | **n=49** | **p-value*** |
| --- | --- | --- |
| Age (years) | 59±10 | 0.635 |
| ***Lifestyle (%)*** |  |  |
| Former smoker | 40.8 | 0.547 |
| Smoker | 25.0 | 0.422 |
| Coffee | 87.8 | 0.698 |
| Alcoholic beverages | 61.2 | 0.655 |
| Occasional physical activity | 38.8 | 0.964 |
| Regular physical activity | 18.4 | 0.078 |
| ***Associated conditions and chronic complications (%)*** |  |  |
| Arterial hypertension | 51.0 | 0.525 |
| Dyslipidemia | 26.5 | 0.051 |
| Coronary heart disease | 8.2 | 0.327 |
| Myocardial infarction | 2.0 | 0.794 |
| Diabetic retinopathy | 12.2 | 0.198 |
| Diabetic nephropathy | 8.2 | 0.341 |
| Diabetic neuropathy | 10.2 | 0.719 |
| ***Use of diabetes medications (%)*** |  |  |
| Sensitizer | 49.0 | **0.007** |
| Insulin secretagogue | 20.4 | 0.995 |
| Insulin | 18.4 | 0.121 |
| Incretin | 12.2 | 0.831 |
| ACE-inhibitors | 24.5 | 0.694 |
| ATII antagonists | 18.4 | 0.343 |
| Beta-blockers | 18.4 | 0.825 |
| Calcium antagonists | 14.3 | 0.922 |
| Diuretics | 16.3 | 0.516 |
| Nitrates | 2.0 | 0.985 |
| Statins | 16.3 | **0.004** |
| Ezetimibe | 0.0 | 0.166 |
| Fibrates | 4.1 | 0.420 |
| Antiplatelet agents | 16.3 | 0.252 |
| Antithrombotics | 8.2 | 0.740 |
| ***Use of ED drugs (%)*** | 18.4 | 0.604 |
| Regular:  *-Sildenafil*  *-Vardenafil*  *-Tadalfil*  *-PGE1*  *-Testosterone* | 2.0  2.0  2.0  0.0  0.0 |  |
| Occasional:  *-Sildenafil*  *-Vardenafil*  *-Tadalfil*  *-PGE1*  *-Testosterone* | 6.1  6.1  2.0  0.0  0.0 |  |
| ***Clinical data*** |  |  |
| Anthropometric parameters |  |  |
| Body-mass index (weight in kg/height in m^2^) | 29.7±5.2 | 0.527 |
| Abdominal circumference (cm) | 103±12 | 0.694 |
| Systolic blood pressure (mm Hg) | 131±18 | 0.634 |
| Diastolic blood pressure (mm Hg) | 80±9 | 0.837 |
| Heart rate (beats per minute) | 77±8 | 0.166 |
| Biochemical parameters |  |  |
| Glycated hemoglobin (%) | 7.5±2.2 | 0.125 |
| Total cholesterol (mg/dl) | 189±36 | 0.521 |
| HDL cholesterol (mg/dl) | 45±12 | 0.648 |
| LDL cholesterol (mg/dl) | 112±37 | 0.745 |
| Triglycerides (mg/dl) | 164±116 | 0.903 |
| Creatinine (mg/dl) | 1.08±0.39 | **0.002** |
| Uricemia (mg/dl) | 5.8±1.4 | 0.418 |
| ALT (U/L) | 26±14 | 0.877 |
| AST (U/L) | 27±20 | 0.890 |
| Total testosterone (ng/ml) | 3.7±2.5 | 0.173 |
| Total testosterone (ng/ml) <2.31 | 31.7 | 0.169 |
| Total testosterone (ng/ml) <3.5 | 53.7 | 0.355 |
| EMAS criteria | 14.6 | 0.599 |
| Depressive symptoms |  |  |
| CES-D score | 19±11 | **0.017** |
| Suspected depression | 24.5 | 0.326 |

EMAS denotes European Male Aging Study; CES-D Center for Epidemiologic Studies Depression Scale.
